# Correcting for tissue nitrogen excretion in multiple breath washout measurements

**DOI:** 10.1371/journal.pone.0185553

**Published:** 2017-10-11

**Authors:** Mica Kane, Jonathan H. Rayment, Renee Jensen, Reginald McDonald, Sanja Stanojevic, Felix Ratjen

**Affiliations:** 1 Division of Respiratory Medicine, Department of Pediatrics, The Hospital for Sick Children, Toronto, ON, Canada; 2 Translational Medicine, The Research Institute, The Hospital for Sick Children, Toronto, ON, Canada; 3 Sidney Kimmel Medical College, Thomas Jefferson University, Philadelphia, PA, United States of America; 4 University of Toronto, Toronto, ON, Canada; University Children`s Hospital Zurich, SWITZERLAND

## Abstract

Nitrogen excreted from body tissues impacts the calculation of multiple breath nitrogen washout (MBW_N2_) outcomes. The aim of this study was to determine the effect of tissue N_2_ on MBW_N2_ outcomes in both healthy subjects and patients with CF and to assess whether it is possible to correct for tissue N_2_. The contribution of tissue N_2_ to MBW_N2_ outcomes was estimated by comparing MBW_N2_-derived functional residual capacity (FRC_N2_) to FRC measured by body plethysmography (FRC_pleth_) and by comparing MBW outcome measures derived from MBW_N2_ and sulfur hexafluoride MBW (MBW_SF6_). Compared to plethysmography and MBW_SF6_, MBW_N2_ overestimated FRC and lung clearance index (LCI). Application of mathematical tissue N_2_ corrections reduced FRC_N2_ values closer to FRC_pleth_ in health and reduced LCI_N2_ in both health and CF, but did not explain all of the differences observed between N_2_-dependent and -independent techniques. Use of earlier washout cut-offs could reduce the influence of tissue N_2._ Applying tissue N_2_ corrections to LCI_N2_ measurements did not significantly affect the interpretation of treatment effects reported in a previously published interventional trial. While tissue N_2_ excretion likely has an impact on MBW_N2_ outcomes, better understanding of the nature of this phenomenon is required before routine correction can be implemented into current MBW_N2_ protocols.

## Introduction

Multiple breath nitrogen washout (MBW_N2_) has been shown to be a feasible and sensitive test to measure ventilation inhomogeneity and detect early obstructive lung disease in children and adults [1,2]. Nitrogen (N_2_) excreted from body tissues through the lungs can impact the calculation of MBW_N2_ outcomes, including the functional residual capacity (FRC) and lung clearance index (LCI) [3,4]. Several studies have measured the elimination of tissue N_2_ in healthy adults from its accumulation during breathing of 100% oxygen for prolonged periods [5–11]. Based on these studies, the tissue N_2_ excretion rate and accumulated volume over time was found to fit a multi-phase exponential curve with the early phases representing the desaturation of highly perfused tissues and the later phases representing the slower desaturation of poorly-circulated and fat-containing tissues. Elimination rates were found to vary both within and between individuals.

Recently, Nielsen *et al*. applied a tissue N_2_ excretion equation to a simulated washout in a two compartment lung model with variable dead space and ventilation heterogeneity [3]. Yammine *et al*. used a different approach to illustrate the effect of tissue N_2_ on the washout by subtracting 1% end-tidal concentration of N_2_ evenly over the course of the washout for one healthy subject and one subject with cystic fibrosis (CF) [4]. These two studies confirmed that there is a greater effect of tissue N_2_ on MBW_N2_ outcomes in disease versus health, but they did not explore whether the contribution of tissue N_2_ can be adequately offset in measurements from subjects with a range of body size and lung disease severity. In patients with CF, increased ventilation inhomogeneity leads to greater washout duration, and in theory, longer washouts have a greater total contribution of tissue N_2_. Therefore, the impact of tissue N_2_ excretion likely introduces greater bias in a subject with significant lung disease compared to a healthy subject of similar size and leads to the overestimation of their FRC and other MBW_N2_ outcomes [2–4,12].

There are limited data to support correcting for the contribution of tissue N_2_; thus it is not currently recommended as per American Thoracic Society/European Respiratory Society (ATS/ERS) consensus statement [12]. As MBW_N2_ develops into an increasingly important clinical research tool for the monitoring of CF lung disease and the assessment of treatment effects, the role of tissue N_2_ must be clarified in order to determine whether it is necessary to correct for its contribution to the MBW_N2_ test. The aim of this study was to estimate the magnitude of tissue N_2_ in both healthy pediatric and adult subjects and patients with CF across a range of disease severity and to assess the effect of applying correction factors for tissue N_2_ on the MBW_N2_ test and on treatment effects in interventional trials.

## Materials and methods

### Study participants

Data were collected as part of four previously published studies [2,13–15]. Healthy participants without a history of respiratory disease or current acute respiratory tract symptoms were recruited from staff and families at the Hospital for Sick Children. Participants with a confirmed diagnosis of CF (defined by a positive newborn screening test or at least one clinical feature of CF in combination with either a documented sweat chloride >60 mEq/L by quantitative pilocarpine iontophoresis test or a genotype with two CF-causing mutations) were recruited from families attending a routine visit to the CF outpatient clinic at the Hospital for Sick Children or St. Michael’s Hospital in Toronto, Canada. Informed written consent was obtained from the participant or parent/guardian for all subjects. The original studies were approved by the Research Ethics Board at the Hospital for Sick Children (REB #1000019945, #1000024909, and #1000023162) and St. Michael’s Hospital (REB #12–139), Toronto, Canada.

### Pulmonary function testing

MBW_N2_ measurements were performed using an open circuit, bias flow system (Exhalyzer D®, EcoMedics AG, Duernten, Switzerland) and associated software (Spiroware® 3.1 EcoMedics AG). A subgroup of subjects also performed MBW tests using a respiratory mass spectrometer system (AMIS 2000, Innovision A/S, Odense, Denmark), which used sulfur hexafluoride (SF_6_) as the tracer gas. MBW_SF6_ traces were analyzed by a single trained observer using custom-written analysis software (TestPoint, Capital Equipment Corp., Billerica, MA, USA). All MBW trials were reviewed for quality control according to guidelines proposed in the ATS/ERS consensus statement [12]. In addition to MBW testing, subjects performed plethysmographic lung volume measurements using the Vmax system (VIASYS CareFusion, San Diego, California, USA) according to ATS standards [16].

### Estimates of tissue N_2_ contribution

FRC measured by body plethysmograph (FRC_pleth_) includes the volume of all compressible intrathoracic gas, whereas only the volume of communicating lung units is measured during MBW. Therefore, in healthy individuals, FRC measured by a gas-dilution technique (such as MBW_N2_) should be equal to or less than that measured by plethysmography [17] in the absence of endogenous production of the tracer gas. Thus the differences between FRC_pleth_ and FRC_N2_ can be used to approximate the contribution of tissue N_2_ to the MBW_N2_. Similarly, as SF_6_ is an exogenous, biologically inert gas that does not dissolve significantly in blood or other tissues, it was used as an indirect reference method to assess the magnitude of the contribution of tissue N_2_ to FRC derived by gas dilution.

### Tissue N_2_ excretion equations

MBW_N2_ assesses ventilation inhomogeneity by examining N_2_ clearance over a series of breaths for the duration of the washout. To generate MBW_N2_ outcomes, the total volume of exhaled gas (net cumulative expired volume; CEV) and the total volume of inert gas expired per breath (cumulative expired volume of N_2_; CEV_N2_) must be measured. FRC and LCI are calculated when Cet_N2_ falls below a predefined threshold (typically 2.5% of the initial CetN2).

$$FRC=\frac{CEV_{N2}}{\left(Cet_{N2,initial}-Cet_{N2,final}\right)}-DS_{pre}$$ Eq 1

$$LCI=\frac{CEV}{FRC}$$ Eq 2

where Cet_N2_ is the end tidal concentration of nitrogen. Cet_N2, initial_ is the end tidal concentration of N_2_ in the first breath of the washout phase, and Cet_N2, final_ is the end tidal concentration of N_2_ in the first breath of the washout phase where Cet_N2_ is less than the target threshold. DS_pre_ is the equipment deadspace proximal to the sampling point of the apparatus.

In order to correct these values for tissue N_2_ excretion, breath-by-breath end tidal body tissue N_2_ concentration (Cet_N2 BT_) as well as the volume of body tissue nitrogen excreted over the washout (V_N2 BT_) are subtracted from Eqs 1 and 2 (Eqs 3–5). The volume of tissue nitrogen was generated for the entire breath (from the start of inhalation to the end of exhalation).

$${Cet}_{N2,BT}=\frac{V_{N2,BTi}}{{VExp}_{i}}$$ Eq 3

$${FRC}_{corr}=\frac{\left({CEV}_{N2}-V_{N2,BT}\right)}{{Cet}_{N2,initial}-{\left({Cet}_{N2}-{Cet}_{N2,BT}\right)}_{final}}-{DS}_{pre}$$ Eq 4

$${LCI}_{corr}=\frac{\left(CEV–V_{N2,BT}\right)}{{FRC}_{corr}}$$ Eq 5

where V_N2,BTi_ is the volume of body tissue nitrogen expired in breath i and VExp_i_ is the net volume of expired gas in breath i. Cet_N2_ is the end tidal concentration of nitrogen. Cet_N2 BT_ is the end tidal concentration of nitrogen derived from the body tissues. Initial subscript indicates the first breath of the washout phase, and final subscript indicates the first breath of the washout phase where (Cet_N2_- Cet_N2 BT_) is less than the target threshold.

**Fig 1:**
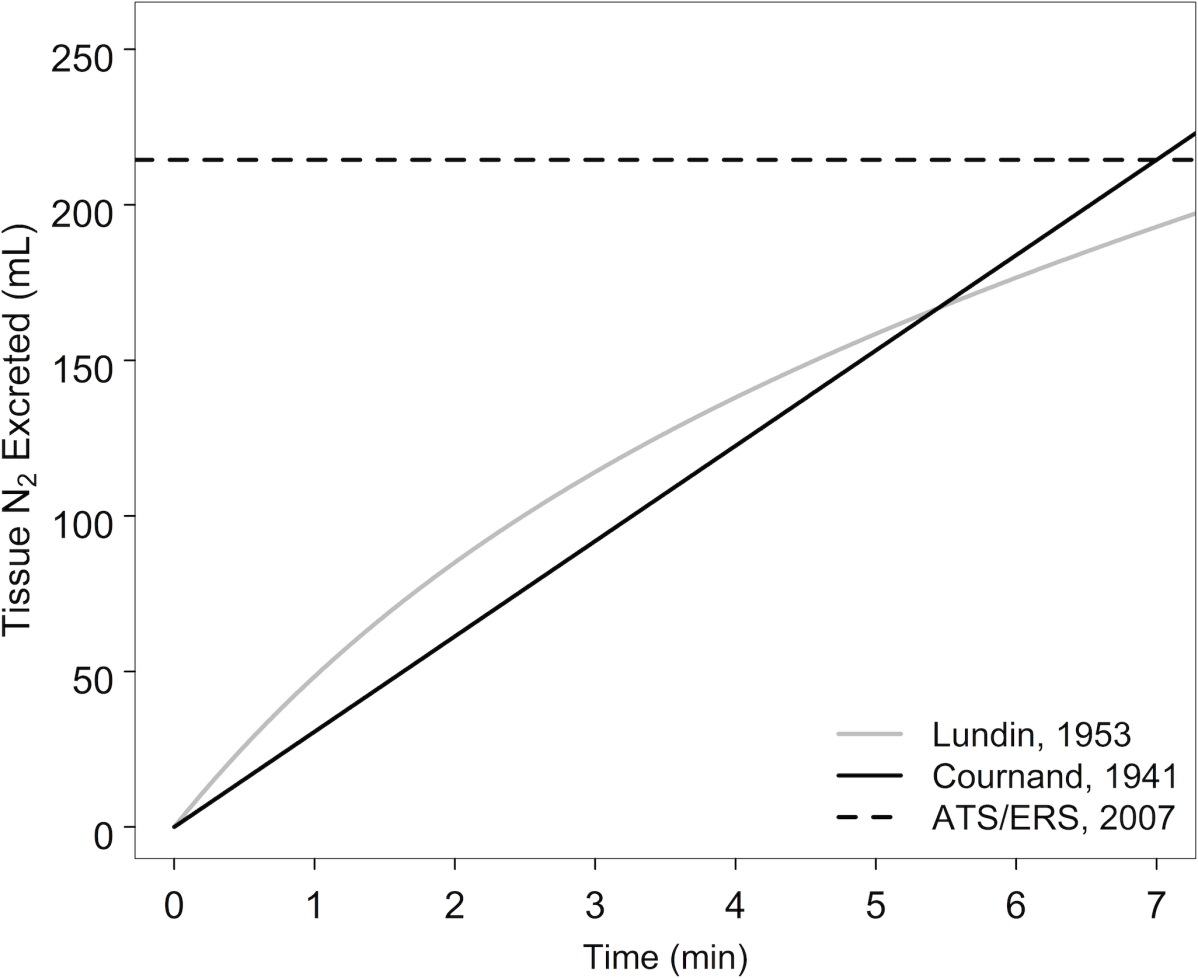
Tissue N_2_ excretion equations used for correction of MBW_N2_ measurements. The three equations used to estimate the volume of N_2_ excreted from the body tissues are plotted over a 7 minute time period. The Cournand 1941 equation was adjusted for a constant excretion rate and plotted for a subject with the average body size of the subjects measured in the Lundin 1953 study. The ATS/ERS equation calculates the volume of tissue N_2_ excreted using Cournand’s 1941 equation standardized to a 7 minute washout for all subjects.

Three different equations (Table 1, Fig 1) were used to derive (V_N2 BT_). Cournand’s body size-dependent Eq (6) and Lundin’s three-phase exponential excretion rate Eq (14) are time-dependent and calculate the end tidal tissue N_2_ concentration (Cet_N2 BT_). The ATS/ERS (22) equation is time-independent and is therefore only used to generate V_N2 BT_ and not Cet_N2 BT_. Therefore, corrected FRC values were generated from all three equations (with Cet_N2, final_ being uncorrected in the ATS/ERS equation), but corrected LCI values were only generated from the time-dependent equations.

**Table 1:** Summary of tissue nitrogen correction equations.

Study (citation)	n	Age range	Equation used
Lundin, 1953 [9]	7	16–42	Rate of excretion (in mL/min) at time *t*: $\frac{dV_{N2}}{dt}={37.3e}^{-0.45t}+{13.9e}^{-0.056t}+{4.82e}^{=0.0054t}$ Integrating to derive *V*_*N*2_ at time *t*: $V_{N2}=\int 37.3e^{-0.45t}+13.9e^{-0.056t}+4.82e^{-0.0054t}dt$ $V_{N2}=\left(\frac{37.3}{0.45}\right)(1-e^{-0.45t})+\left(\frac{13.9}{0.056}\right)(1-e^{-0.056t})+\left(\frac{4.82}{0.0054}\right)(1-e^{-0.0054t})$ Where t = time in minutes
Cournand, 1941 [6]	30	9–44	$V_{N2}=\frac{t}{420}\times [(96.5\times BSA)+35]$ Where t = time in seconds
ATS/ERS [16]	NA	NA	*V*_*N*2_ = (96.5 × *BSA*) + 35

$BSA=Body\;Surface\;[m^{2}]=\frac{{Weight[kg]}^{0.425}\times {Height[cm]}^{0.725}\times 71.84}{10000}$

VN_2_ = excreted volume of nitrogen (mL)

NA = Not applicable

To assess whether the breath-by-breath calculated FRC achieves a plateau, linear regression slopes of the FRC_N2_/time curves were calculated for the second half all uncorrected and corrected washouts.

Comparisons of the corrected and uncorrected FRC and LCI results were made with FRC_pleth_ and the difference in FRC and LCI measured by MBW_N2_ and MBWSF_6_, when available. FRC and LCI values were also re-calculated from the Cournand and Lundin-corrected measurements at the standard MBW end-point of 2.5% normalized end-tidal N_2_ concentration, as well as for earlier end-points of 5%, 9%, 12%, and 18% normalized end-tidal N_2_ concentration. These end-points were chosen to reflect previous studies that evaluated earlier cut-offs and existing software algorithms [18].

### Accuracy of derived nitrogen concentration

Since N_2_ concentration values generated by the Exhalyzer D are derived from O_2_ and CO_2_ concentrations and not directly measured, our results may be biased if these derived values are inaccurate_._ To ensure the accuracy of the derived N_2_ values over the range observed during a MBW_N2_ test, we compared the C_ET_ N_2_ calculated by the Spiroware software to a set of reference gases generated by blending medical air (compressed on site with presumed gas concentrations: F_1_CO_2 =_ 0.0004, F_1_O_2_ = 0.2095, F_1_N_2_ = 0.7808, F_1Ar_ = 0.0093) with a high precision gas mixture (F_2_CO_2 =_ 0.0500, F_2_O_2_ = 0.9500; Praxair Canada, Mississauga ON). FN_2_ of the mixed reference gas (F_M_N_2_) was calculated using Dalton’s Law of partial pressures, the fractional concentrations of the reference gases and the measured FO_2_ of the mixed gas (F_M_O_2_)_._

F_M_O_2_ was measured using the Oxigraf laser oxygen analyzer (Oxigraf Inc, Sunnyvale CA, USA) within the Exhalyzer D^®^. The accuracy of the Oxigraf analyzer was confirmed against a paramagnetic oxygen analyzer (Servomex 570A, Servomex, Sugar Land TX, USA). The reported FN_2_ from the Exhalyzer D^®^ was compared to F_M_N_2_ over the range of FN_2_ observed in a washout (0.01–0.8).

### Statistical analysis

Study population characteristics and lung function measurements were summarized as mean and standard deviation (SD). Group differences were calculated using two-sample t-tests, whereas differences in outcomes within the same subject were compared using paired t-tests. The agreement between outcomes within the same subject was assessed using Bland-Altman plots. Pearson correlations were used to determine the correlation between two outcomes. All statistical analysis was conducted using R version 3.1.2 (R Foundation for Statistical Computing, Vienna, Austria).

## Results

### Accuracy of derived nitrogen concentration

The absolute difference between FN_2_ reported by the Exhalyzer D and the reference concentrations (F_M_N_2_) was measured over the full range of washout nitrogen concentrations. The mean absolute difference was 0.064% (95% CI -0.032 to 0.16). All measured differences (n = 14) were less than 0.12%. Therefore, we considered the CetN_2_ derived by the Exhalyzer D to accurately reflect the true CetN_2_.

### Estimates of tissue N_2_ contribution to FRC

Characteristics of study participants included are shown in Table 2. Healthy subjects and individuals with CF did not differ in age or lung volumes measured by either MBW_N2_ or body plethysmography. As expected, LCI measured by MBW_N2_ was significantly higher in patients with CF.

**Table 2:** Characteristics of study participants. Values are presented as mean (SD) unless otherwise indicated. P value indicates group difference between health and CF.

	Health (n = 43)	CF (n = 35)	Mean difference (95% CI)	P-value
Age (years)	16.5 (5.5)	16.2 (8.3)	0.3 (-2.9 to 3.6)	0.83
Females (%)	60.5	60.0	0.5 (-21 to 22)	0.96
Height (cm)	163.0 (15.8)	157.5 (16.8)	5.5 (-1.9 to 12.9)	0.14
Weight (kg)	57.8 (20.6)	51.7 (17.0)	6.1 (-2.4 to 14.6)	0.16
FRC_pleth_ (L)	2.29 (0.88)	2.49 (1.06)	-0.2 (-0.72 to 0.33)	0.47
FRC_N2_ (L)	2.59 (0.92)	2.46 (0.96)	0.13 (-0.31 to 0.55)	0.58
LCI	6.88 (0.49)	12.04 (3.60)	-5.16 (-6.40 to -3.91)	<0.001

**Fig 2:**
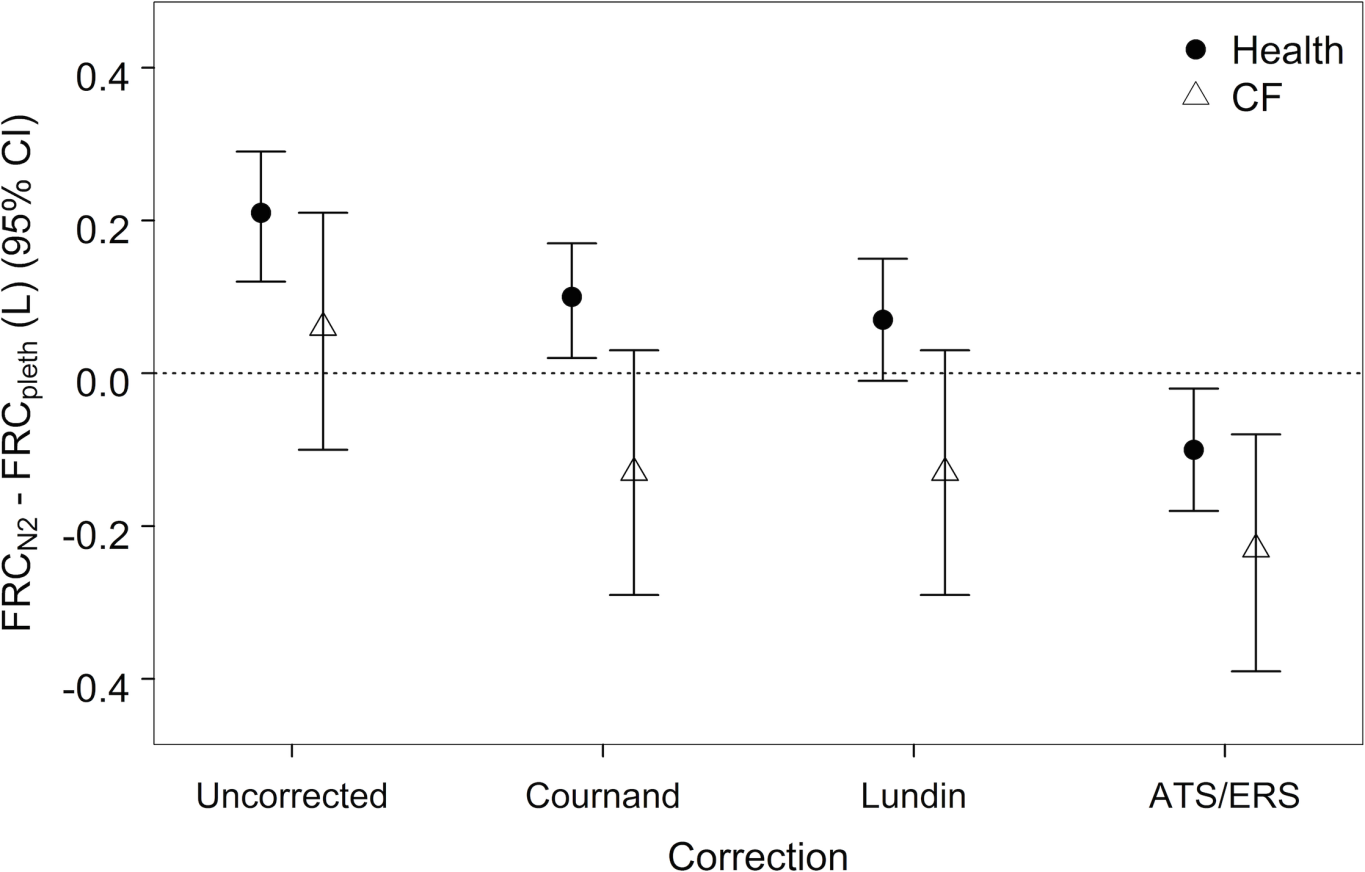
Within-subject difference in FRC measured by multiple breath nitrogen washout (FRC_N2_) and body plethysmography (FRC_pleth_). The mean within-subject difference FRC_N2_—FRC_pleth_ decreased after the three tissue N_2_ excretion equations were applied to washout data from healthy and CF populations.

FRC measured by the MBW_N2_ gas dilution technique (FRC_N2_) should be smaller than or equal to, but not exceed, FRC measured by body plethysmography (FRC_pleth_). However, healthy subjects who performed both techniques had FRC_N2_ values that were on average greater than FRC_pleth_ (mean difference 0.21L; 95% CI 0.12 to 0.29, p<0.001). In contrast, the relationship between FRC_N2_ and FRC_pleth_ was inconsistent in subjects with CF (mean difference 0.06; 95% CI -0.10 to 0.21, p = 0.44). FRC_N2_ values were recalculated by applying the three tissue N_2_ excretion equations. Application of all three tissue N_2_ excretion equations decreased FRC_N2_ values compared to FRC_pleth_ in health and CF (Fig 2).

Given that the Cournand and Lundin excretion equations improve the FRC_N2_ agreement with plethysmography, the uncorrected FRC_N2_ (FRC_uncorr_) and the FRC_N2_ corrected (FRC_Cournand_ and FRC_Lundin_) were then compared within subjects (Table 3).

**Table 3:** Estimates of tissue N_2_ contribution to MBW outcomes at the 2.5% washout cut-off. Values are presented as the mean within-subject difference (95% CI) of the uncorrected–corrected MBW_N2_ outcome. Outcomes were corrected by applying either the Cournand or Lundin tissue N_2_ excretion equations.

Outcome	Health *Mean difference (95% CI)*	CF *Mean difference (95% CI)*
FRC_N2_ (L)		
Cournand	0.11 (0.10; 0.13), p<0.001	0.18 (0.15; 0.21), p<0.001
Lundin	0.13 (0.12; 0.15), p<0.001	0.19 (0.17; 0.20), p<0.001
CEV_N2_ (L)		
Cournand	1.63 (1.36; 1.90), p<0.001	4.41 (3.30; 5.52), p<0.001
Lundin	1.57 (1.37; 1.76), p<0.001	3.22 (2.40; 4.03), p<0.001
LCI_N2_		
Cournand	0.35 (0.29; 0.42), p<0.001	0.90 (0.63; 1.17), p<0.001
Lundin	0.30 (0.23; 0.36), p<0.001	0.41 (0.17; 0.65), p = 0.001

**Fig 3:**
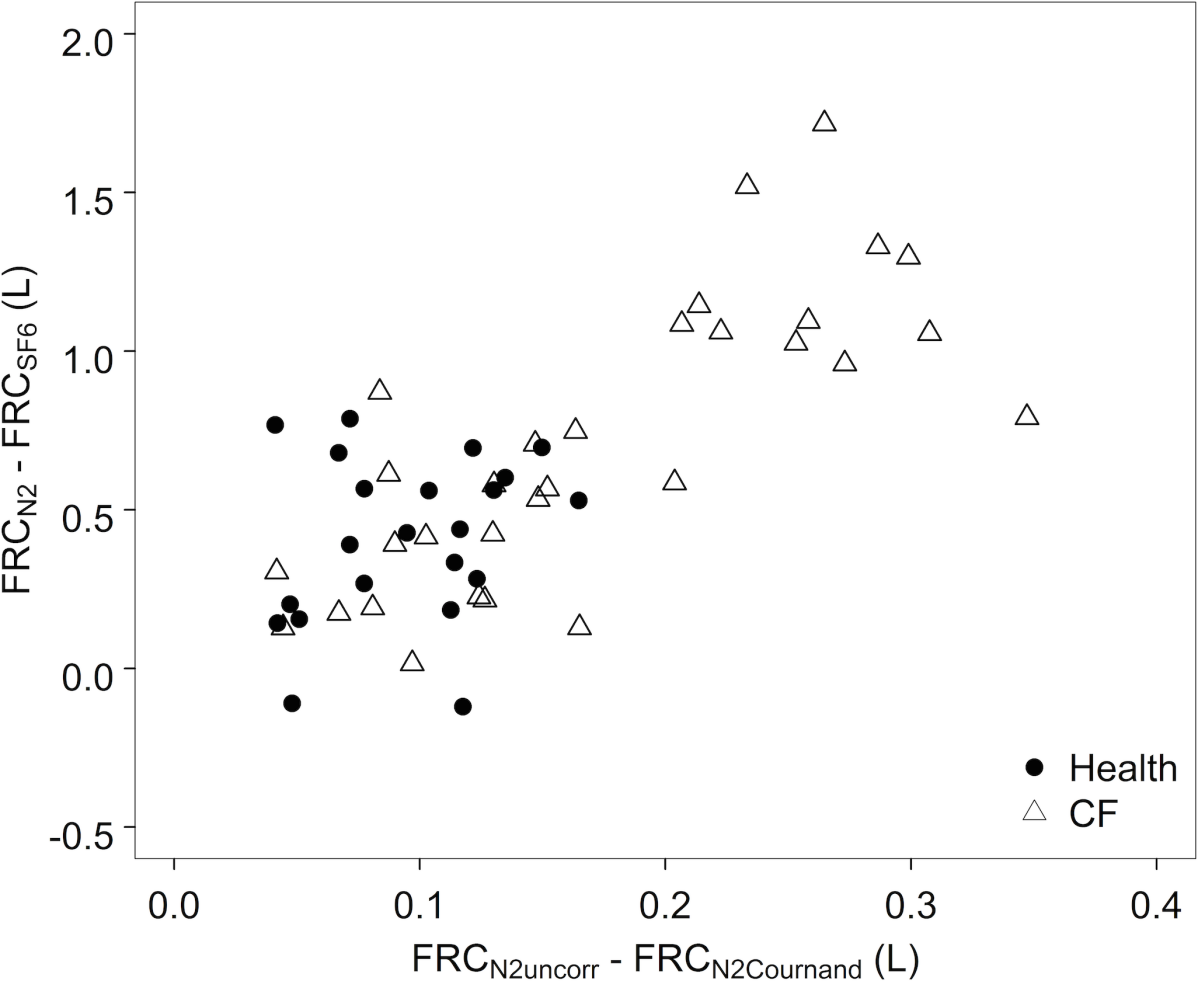
Relationship between the contribution of tissue N_2_ to FRC_N2_ and the difference between FRC as measured by MBW_N2_ and MBW_SF6_. FRC_N2_ became disproportionately greater than FRC_SF6_ as the contribution of tissue N_2_ estimated by the within-subject difference FRC_N2_—FRC_Cournand_ increased.

The within-subject difference in FRC as measured by MBW_N2_ and MBW_SF6_ (FRC_N2_ –FRC_SF6_) were also compared with the estimated contribution of tissue N_2_ to FRC_N2_. The difference between FRC_N2_ and FRC_SF6_ was positively correlated with increased washout time (r = 0.69, p<0.001). FRC_N2_ became disproportionately larger than FRC_SF6_ as the contribution of tissue N_2_ as estimated by FRC_uncorr_−FRC_Cournand_ increased (r = 0.68, p<0.001) (Fig 3).

**Fig 4:**
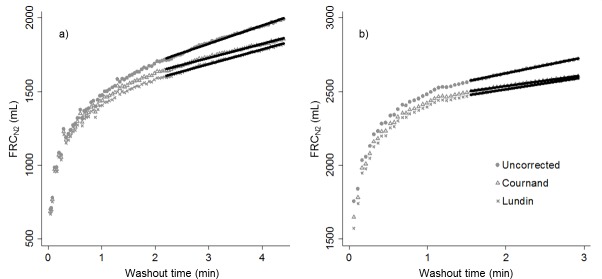
**Representative examples of FRC_N2_ plotted against washout time in a) a healthy subject and b) a patient with CF.** Application of the Cournand and Lundin tissue N_2_ excretion equations resulted in a less pronounced increase in the calculation of FRC_N2_ over the course of the washout, but a plateau was never achieved. Linear regressions of these curves over the second half of the washout are shown, demonstrating the slopes that are reported in Table 4.

When plotted against washout time, the breath-by-breath calculation of FRC_N2_ did not plateau as would be expected in a closed system, but rather continued to increase throughout the washout (representative examples from health and CF shown in Fig 4). This is consistent with continuous tissue N_2_ excretion. Breath-by-breath correction of the FRC_N2_ values using the Cournand and Lundin equations decreased the rate of rise of the FRC_N2_ by 23–34%, but did not reduce it to zero (Fig 4, Table 4). The absolute and relative magnitudes of the decrease in the FRC/time slope were greater in healthy subjects than in those with CF for both the Lundin and Cournand equations (Table 4).

**Table 4:** Average slopes of the second half of all uncorrected, Lundin-corrected and Cournand-corrected FRC_N2_/breath number curves (depicted graphically in Fig 4) for healthy subjects and those with CF. Average paired difference (uncorrected-corrected) in absolute and relative (percent of uncorrected slope) terms are shown. Data are expressed as mean 95% confidence interval) unless otherwise stated.

	Health	CF
Uncorrected slope (mL/min)	104.2 (97.9, 110.6)	125.3 (115.4, 135.2)
Corrected slope (Lundin)	77.4 (71.4, 83.5)	100.4 (90.1, 110.7)
Absolute diff (Lundin)	26.8 (25.2, 28.4)	24.9 (23.4, 26.4)
Relative diff (Lundin) (%)	27.4 (25.0, 29.9)	22.5 (19.6, 25.3)
Corrected slope (Cournand)	70.5 (65.0, 76.0)	94.2 (84.9, 103.5)
Absolute diff (Cournand)	33.7 (32.3, 35.1)	31.1 (29.6, 32.6)
Relative diff (Cournand) (%)	33.9 (31.9, 35.8)	26.7 (24.6, 28.8)

### Estimates of tissue N_2_ contribution to CEV and LCI

Similar to FRC_N2_, application of tissue N_2_ excretion equations to MBW_N2_ data resulted in lower CEV_N2_ and LCI_N2_ values (Table 2). Application of the Cournand excretion equation shortened the washout by an average of 2.9 breaths in health (95% CI 2.5 to 3.3, p<0.001) and 7.6 breaths in CF (95% CI 6.3 to 8.8, p<0.001). Similar results were observed for the Lundin equation (2.9 and 5.9 breaths in health and CF, respectively). Since the ATS/ERS correction is not time-dependent and only corrects FRC_N2_ for the contribution of tissue N_2_, it was not used to correct LCI and CEV values.

When LCI as measured by MBW_N2_ and MBW_SF6_ were compared within subjects (LCI_N2_ –LCI_SF6_), LCI_N2_ became disproportionately greater than LCI_SF6_ as disease severity (LCI_N2_) increased (r = 0.53, p<0.001). Similar to FRC_N2_, there was a significant and positive correlation observed between LCI_N2_ –LCI_SF6_ and the effect of tissue N_2_ as estimated by LCIuncorr−LCI_Cournand_ (r = 0.55, p<0.001).

### Impact of tissue N_2_ at earlier washout cut-offs

**Fig 5:**
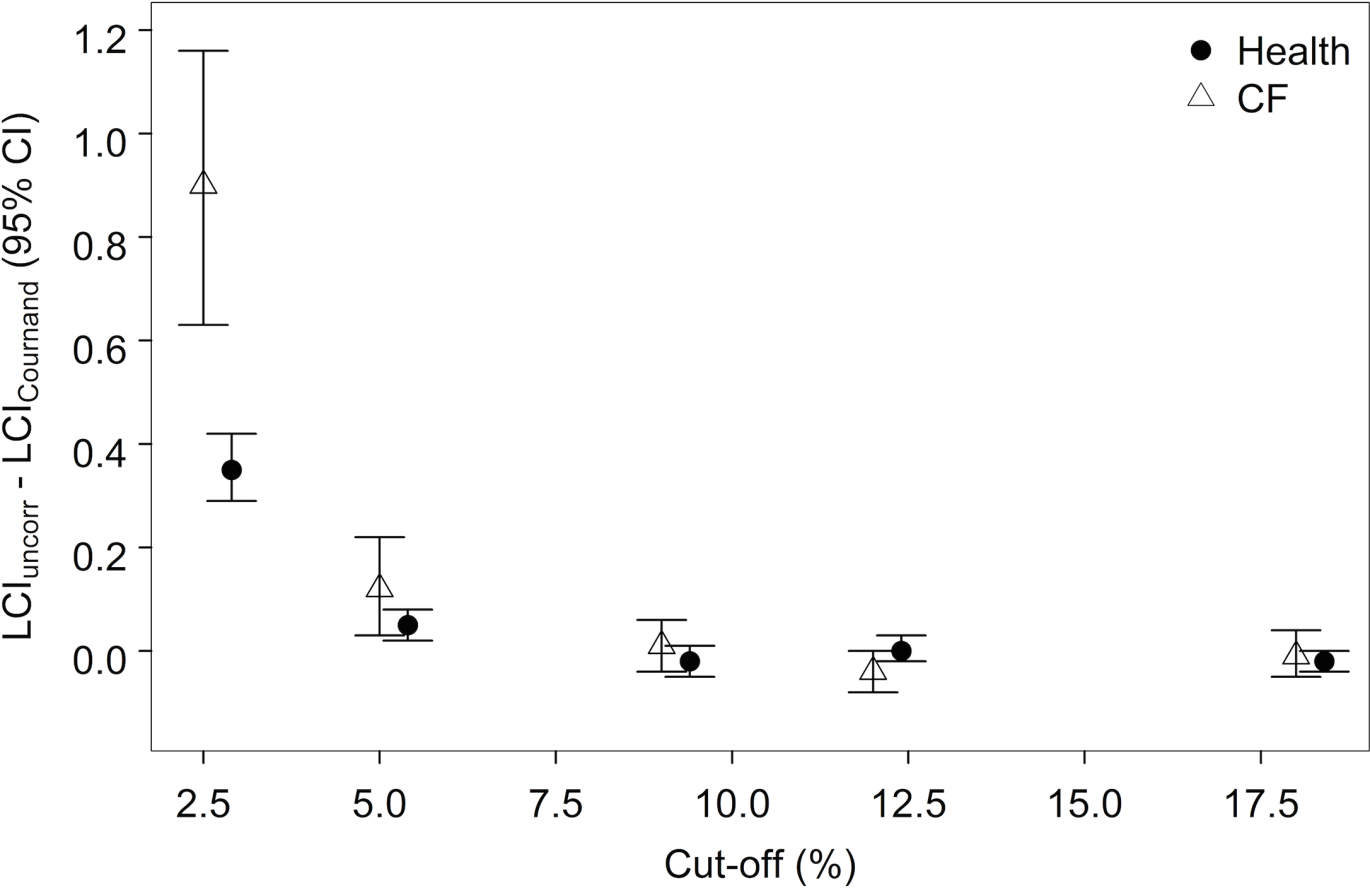
Within-subject difference between uncorrected LCI_N2_ and LCI_N2_ corrected for tissue N_2_ excretion (LCI_Cournand_) at different washout cut-offs. The difference was progressively smaller when calculated at earlier cut-offs of the washout in both health and CF.

**Fig 6:**
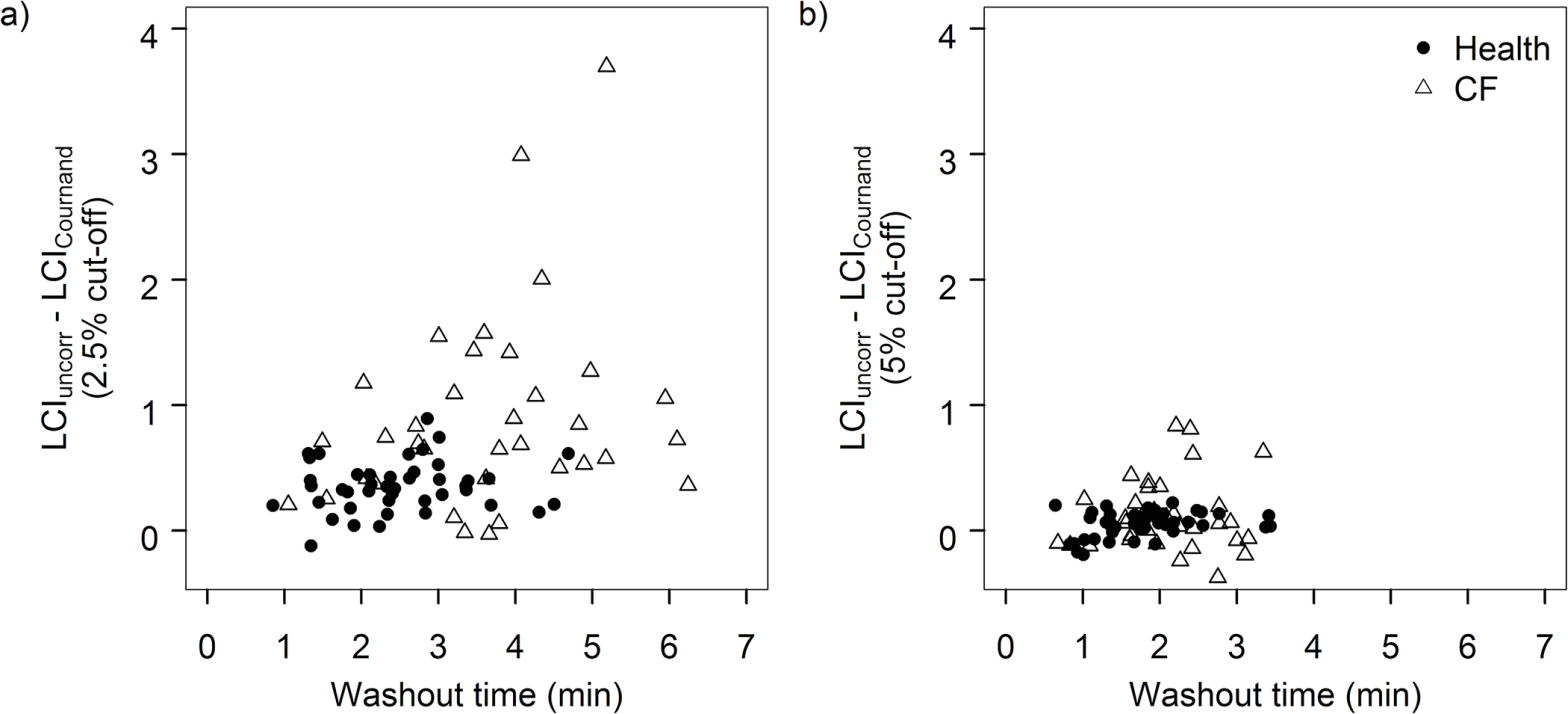
**Relationship between the contribution of tissue N_2_ to LCI_N2_ and length of washout calculated at a) the traditional 2.5% washout cut-off and b) the 5% washout cut-off.** The contribution of tissue N_2_ to LCI_N2_ calculated at the 2.5% cut-off (LCI_uncorr_−LCI_Cournand_) increased as washout time increased. However, this relationship was no longer observed at the earlier 5% cut-off.

With application of the Cournand tissue N_2_ excretion equation, the effect of tissue N_2_ (LCI_uncorr_−LCI_Cournand_) decreased when LCI_N2_ was calculated at earlier cut-offs of the washout (Fig 5). Compared to the traditional cut-off of 2.5% normalized end-tidal concentration of N_2_, the difference between corrected and uncorrected LCI (LCI_uncorr_−LCI_Cournand_) was less pronounced at the 5% cut-off and was no longer significant by the 9% cut-off. While the effect of tissue N_2_ (LCI_uncorr_−LCI_Cournand_) on LCI_N2_ calculated at the 2.5% cut-off increased as disease severity (LCI_N2_) increased (r = 0.61, p<0.001) (Fig 6A), this relationship was not observed at the 5% cut-off (r = 0.17, p = 0.13) (Fig 6B).

### Impact of tissue N_2_ correction on interventional trial outcomes

Both the Cournand and Lundin equations were applied to MBW data of an observational study investigating the effect of ivacaftor on LCI in children with class 3 mutations in CF [14] (Table 5). The Lundin-corrected treatment effect was significantly smaller than the uncorrected value (p = 0.01) and the Cournand-corrected difference showed a similar trend (p = 0.11). This change in treatment effect was driven by a greater negative correction in pre-treatment LCI than post-treatment LCI by both Lundin (pre-treatment correction -0.9 [-1.3, -0.5] units; post-treatment correction -0.6 [-0.8, -0.3] units) and Cournand (pre-treatment correction -1.3 [-1.8, -0.8] units; post-treatment correction -0.9 [-1.3, -0.4] units) equations. Neither correction equations changed the direction or significance of the treatment effect.

**Table 5:** Effect of applying Lundin and Cournand correction equations to previously published observational MBW data. Data are shown as pre-treatment and post-treatment LCI with paired treatment effect. Values are presented as mean (SD) unless otherwise indicated.

	Pre-treatment LCI	Post-treatment LCI	Treatment effect mean difference (95%CI)
Ivacaftor [14]			
Uncorrected	13.7 (3.7)	11.6 (4.1)	-2.2 (-3.0, -1.3)
Lundin	12.8 (3.8)	11.0 (3.9)	-1.8 (-2.6, -0.9)
Cournand	12.4 (3.6)	10.7 (3.6)	-1.8 (-2.8, -0.7)

## Discussion

In agreement with previous studies, these data suggest that excretion of N_2_ from body tissues affects MBW_N2_ outcomes. The effects of tissue N_2_ are greater in patients with longer washouts. This contribution of tissue N_2_ to FRC_N2_ and LCI_N2_ is less pronounced at earlier cut-offs of the washout. Application of correction equations for tissue N_2_ significantly reduced, but did not completely eliminate, the effect of tissue N_2_ on MBW_N2_ outcomes. Importantly, application of these tissue N_2_ correction equations did not significantly alter treatment effects previously observed in interventional trials. Thus, while the excretion of tissue N_2_ has a measurable effect on MBW_N2_ outcomes, correction for tissue N_2_ using currently available approaches cannot be recommended at the present time.

FRC is an integral component of the calculation of LCI by MBW and therefore a reliable FRC is required to derive a reliable LCI. While there is no gold standard for the determination of FRC, body plethysmography and inert gas washout are the most commonly used techniques [16,17]. In the current study, FRC_N2_ was compared to FRC_pleth_ and FRC_SF6_ to estimate the contribution of tissue N_2_ excretion. With FRC_pleth,_ the volume of all compressible intrathoracic gas is measured whereas only the volume of communicating lung units is measured with FRC_N2_. Therefore, FRC measured by gas-dilution technique (such as MBW_N2_) should be equal to or less than that measured by plethysmography in the absence of endogenous production of the tracer gas [17]. FRC_SF6_ is also calculated using a gas-dilution technique, and because it is an exogenous, biologically inert gas that does not dissolve significantly in blood or other tissues, it was used as comparator to assess for the contribution of tissue N_2_ excretion to FRC_N2_.

We found that FRC_N2_ was systematically overestimated compared to FRC_SF6_ and, to a more variable extent, FRC_pleth_ (Figs 2 and 5). This suggests that there is a systematic difference between these tests and that the observed differences were not entirely due to intrinsic differences between the MBW and plethysmographic techniques. While our analyses focused on the potential effect of tissue nitrogen excretion on this overestimation, there are other explanations for this disparity that could contribute to the observed differences that were not assessed in the current study, such as testing order, technical inconsistencies in the MBW equipment, and physical differences between SF_6_ and N_2_ tracer gases.

The order of tests could have inadvertently biased the results through effects of tissue hysteresis or other unknown mechanisms. In the original study, all plethysmographic testing was performed after the MBW testing and the order of MBW_SF6_ and MBW_N2_ was randomized [2]. All MBW-based outcomes can be affected by errors in gas concentration measurement, flow-gas signal alignment, dead-space correction and other device-specific settings [19–21]. In this study, we used working-group recommended equipment and software settings on both the Exhalyzer D and AMIS 2000 devices and applied standardized quality control criteria to each MBW trial. We also confirmed the accuracy of the N_2_ concentration calculation (as FN_2_ is derived from measured O2 and CO_2_ concentrations using the Exhalyzer device) across a range of gas standards. Despite our attempts to minimize technical software or device-specific inconsistencies, these cannot be completely ruled out as sources of systematic error that could contribute to the discrepancies observed.

The intrinsic properties of MBW_SF6_ and MBW_N2_ tests could also have contributed to these differences. The molecular properties of SF_6_ and N_2_ likely result in differences in their diffusion-convection fronts, which could potentially impact MBW outcomes [22]. MBW_SF6_ requires a wash-in equilibration phase as SF_6_ is an exogenous tracer gas, and while standardized quality control techniques were implemented to attempt to ensure complete SF_6_ washing, it is possible that incomplete wash-in of the SF_6_ could result in altered excretion kinetics. Finally, the 100% oxygen washout phase in MBW_N2_ could also theoretically have pro-atelectatic effects, thus altering pulmonary gas flow dynamics. While simultaneous direct measurements of N_2_ and SF_6_ on the same device would permit an ideal comparison of these two MBW systems, unfortunately, high O_2_ concentration impairs the ability of the AMIS 2000 respiratory mass spectrometer to measure N_2_ concentrations and can therefore not be used to measure the two gases in the context of a 100% oxygen washout. Overall, our results need to be interpreted in the context of these potential limitations; nevertheless, the consistent overestimation of FRC_N2_ when compared to FRC_pleth_ and FRC_SF6_ suggests that tissue N_2_ likely contributes to this phenomenon.

Both FRC_N2_ and LCI_N2_ decreased significantly upon application of the tissue N_2_ excretion equations in both healthy subjects and subjects with CF, with greater differences observed in CF. The estimates of the contribution of tissue N_2_ to FRC_N2_ and LCI_N2_ are similar to those previously predicted by a two-compartment lung model including variable ventilation heterogeneity and dead space effects [3]. However, the difference between FRC_SF6_ and FRC_N2_ was significantly greater than the degree of correction applied by either Lundin or Cournand equations (Fig 3). Also, application of the correction equations only decreased the time-dependent-rise in FRC_N2_ by ~30% (Fig 4; Table 4). These findings suggest either that the equations used in this study underestimate the amount of tissue N_2_ excretion, or that there are other factors in addition to tissue N_2_ secretion that are driving this difference.

The Lundin tissue N_2_ excretion equation is based on the average of measurements derived from healthy adults, therefore its application to MBW_N2_ data derived from subjects of varying size is limited. Compared to the Cournand equation, which was derived from subjects ranging from 9 to 44 years old and adjusts for a subject’s body size, the Lundin equation may overestimate the effect of tissue N_2_ excretion in smaller pediatric subjects. Although the Cournand equation may introduce less error overall in MBW_N2_ measurements from subjects with a range of body size, it assumes a constant rate of N_2_ excretion from the body tissue which is unlikely to be the case in subjects of varying body composition and between health and disease. In a recently published study [23], the rate of tissue N2 excretion was simultaneously performed on MBWN2 and MBWSF6 washouts and confirmed the time-dependent nature of tissue N2 excretion and demonstrated higher rates of tissue N2 excretion during moderate exercise.”

Ideally, direct measurement of pulmonary N_2_ excretion of tissue N_2_ with modern equipment across a range of ages, body compositions and disease states would allow us to generate an optimal correction equation. However, due to the long duration of the testing and uncomfortable testing setup, replications of these early studies would be extremely challenging to conduct today, especially in children [6,9]. Furthermore, no mathematical correction for tissue N_2_ excretions will be ideal for several reasons. First, even with modern technology, it is impossible to precisely isolate all of the N_2_ in the lungs that was excreted from the body tissue, especially during the beginning of the washout when the relative proportion is very small; the derived equations are reflections of this imprecision. Second, the contribution of N_2_ from the body tissue is likely dependent not only on time and body size, but also on factors such as cardiac output, tissue perfusion, body fat content, ventilation homogeneity, and dead space [3,6,8,23–26]. Any number of these physiological factors could be altered in a disease like CF and could confound the estimation of tissue N2 excretion.

The extent to which the MBW_N2_ outcomes diverged from both MBW_SF6_ and MBW_pleth_ was related to the length of the washout. This correlation makes intuitive sense, since individuals with longer washouts (greater ventilation inhomogeneity) spend a longer time at lower end-tidal N_2_ concentrations, thereby accentuating the relative contribution of excreted tissue nitrogen. Given this finding, we showed that the contribution of tissue N_2_ can be minimized by calculating MBW_N2_ outcomes at earlier cut-offs of the washout, such as at the 5% normalized end tidal concentration of N_2_. Using an earlier cut-off of the washout has the additional benefit of shortening the total time it takes to perform an MBW test; however, there is some evidence that there may be a trade-off with decreased sensitivity to treatment efficacy [18]. Nevertheless, the use earlier cut-off for MBW_N2_ did not affect the significance of treatment effects observed in a study of Ivacaftor treatment [14], suggesting that the sensitivity of an MBW cut-off may depend upon the effect size of the intervention. The optimal MBW_N2_ cutoff for interventional studies may depend on study design and treatment.

Finally, to address the practical question of whether or not the correction for tissue N_2_ excretion could affect the results of previously reported interventional studies, we applied tissue N_2_ correction equations to raw MBW_N2_ data from a study that assessed the effect of ivacaftor on LCI [14]. Overall, this study had a large treatment effect (-2.2 LCI units) and we found that applying tissue N_2_ correction equations attenuated the treatment response, but did not change the significance or direction of the treatment effect. This attenuation of the treatment response occurred primarily by reducing the post-treatment LCI by a greater amount than the pre-treatment LCI and is likely a reflection of the observation that tissue N_2_ has a greater contribution in longer washouts. Taken together, these results suggest that non-correction for tissue N_2_ release may result in marginally overestimated treatment effects. While this does not significantly affect the results of the studied trial, it is conceivable that smaller treatment effects could be amplified by non-correction for tissue N_2_.

In conclusion, MBW_N2_ outcomes are systematically different from MBW_SF6_ and plethysmography. We show that correction for tissue N_2_ excretion using previously derived equations can reduce, but not eliminate, these differences. This suggests that either there are other physiologic/experimental factors contributing to this difference, or that the correction equations that were used underestimate the quantity of tissue N_2_ excretion. Given our data, we suggest that there is currently inadequate knowledge of the true rate of pulmonary tissue nitrogen excretion to suggest a standard correction equation for this phenomenon in the calculation of MBW outcomes. Further study (ideally simultaneous MBW_N2_ and MBW_SF6_ measurements using an appropriately tuned mass spectrometer) could elucidate the contribution of tissue N_2_ to MBW_N2_ outcome measures. Until this is clarified, it should be recognized that the magnitude of treatment responses measured with MBW_N2_ may be over-estimated by tissue N_2_ excretion, however, application of correction equations in this study did not change the direction or significance of the treatment effects of a previously studied intervention.
